# Bioinformatics analysis and *in vivo* validation of ferroptosis-related genes in ischemic stroke

**DOI:** 10.3389/fphar.2022.940260

**Published:** 2022-11-24

**Authors:** Chang Liu, Zhixi Li, Hongjie Xi

**Affiliations:** ^1^ Heilongjiang Province Key Laboratory of Research on Anesthesiology and Critical Care Medicine, Harbin, China; ^2^ The Key Laboratory of Myocardial Ischemia Organization, Chinese Ministry of Education, Harbin, China; ^3^ Department of Anesthesiology, The Second Affiliated Hospital of Harbin Medical University, Harbin, China

**Keywords:** ischemic stroke, ferroptosis, HIF-1 signaling pathway, dexmedetomidine, microarray, oxidative stress, inflammatory response

## Abstract

Ischemic stroke (IS) is a neurological condition associated with high mortality and disability rates. Although the molecular mechanisms underlying IS remain unclear, ferroptosis was shown to play an important role in its pathogenesis. Hence, we applied bioinformatics analysis to identify ferroptosis-related therapeutic targets in IS. IS-related microarray data from the GSE61616 dataset were downloaded from the Gene Expression Omnibus (GEO) database and intersected with the FerrDb database. In total, 33 differentially expressed genes (DEGs) were obtained and subjected to functional enrichment and protein–protein interaction (PPI) network analyses. Four candidate genes enriched in the HIF-1 signaling pathway (*HMOX1*, *STAT3*, *CYBB*, and *TLR4*) were selected based on the hierarchical clustering of the PPI dataset. We also downloaded the IR-related GSE35338 dataset and GSE58294 dataset from the GEO database to verify the expression levels of these four genes. ROC monofactor analysis demonstrated a good performance of *HMOX1*, *STAT3*, *CYBB*, and *TLR4* in the diagnosis of ischemic stroke. Transcriptional levels of the above four genes, and translational level of GPX4, the central regulator of ferroptosis, were verified in a mouse model of middle cerebral artery occlusion (MCAO)-induced IS by qRT-PCR and western blotting. Considering the regulation of the HIF-1 signaling pathway, dexmedetomidine was applied to the MCAO mice. We found that expression of these four genes and GPX4 in MCAO mice were significantly reduced, while dexmedetomidine reversed these changes. In addition, dexmedetomidine significantly reduced MCAO-induced cell death, improved neurobehavioral deficits, and reduced the serum and brain levels of inflammatory factors (TNF-α and IL-6) and oxidative stress mediators (MDA and GSSG). Further, we constructed an mRNA-miRNA-lncRNA network based on the four candidate genes and predicted possible transcription factors. In conclusion, we identified four ferroptosis-related candidate genes in IS and proposed, for the first time, a possible mechanism for dexmedetomidine-mediated inhibition of ferroptosis during IS. These findings may help design novel therapeutic strategies for the treatment of IS.

## Introduction

As the third leading cause of death and the first leading cause of disability, stroke is a major public health concern worldwide (Tao et al., 2020). Ischemic stroke (IS) results from cerebral hypoperfusion and occupies roughly 85% of stroke cases (Liu et al., 2020). Although endovascular-reperfusion therapy can improve outcomes in some patients, the prognosis of IS still poor due to the narrow therapeutic window, potential hemorrhagic risk, and subsequent reperfusion injury (Jahan et al., 2019; Liao et al., 2020).

Multiple intricate biological processes have been implicated in IS-related neuronal death. These include oxidative stress, neuroinflammation, excitotoxicity, and various forms of regulated cell death (Zhang et al., 2019; Han et al., 2020; Liao et al., 2020; Liu et al., 2020). Among the latter, ferroptosis constitutes a form of programmed necrosis that shows unique features, namely iron dependency and accumulation of reactive oxygen species (ROS) (Gao et al., 2016). Since its identification, ferroptosis has been reported in several neurological disorders, including cerebral ischemia/reperfusion injury (CIRI) and neurodegenerative diseases (Yan et al., 2021). Researchers reported that disturbance in the levels of trace elements, including iron, combined with a reduction in antioxidant activity, associated with the etiology of CIRI (Fang et al., 2013). Supporting evidence for this association was provided by the fact that specific interventions could reverse the increased level of lipid peroxide and the reduction of ferroptosis marker glutathione peroxidase 4 (Guan et al., 2019). However, the specific molecular mechanism underlying ferroptosis in IS poorly understood.

Dexmedetomidine (DEX), an α2 receptor antagonist, has been shown to significantly reduce both the inflammatory response and oxidative stress in diseases caused by ischemia-reperfusion injury, potentially via the HIF-1 signaling pathway (Shi et al., 2021). However, studies investigating the specific mechanisms by which DEX attenuates ferritin formation during CIRI are still lacking.

In the present study, we used publicly available gene expression datasets and bioinformatics analysis to identify critical genes and possible mechanisms related to ferroptosis in IS. By analyzing GEO microarray expression profiles from control and IS rat brains and intersecting these data with the FerrDB database, we identified and conducted functional enrichment analyses on ferroptosis-related differentially expressed genes (DEGs). After validation of the expression and diagnostic efficacy of these four genes in different datasets, the effect of DEX on ferroptosis and these four genes in the HIF-1 signaling pathway during IS was assessed in a mouse model of IS induced by middle cerebral artery occlusion (MCAO). Finally, we constructed an mRNA-miRNA-lncRNA interaction network based on main ferroptosis-related candidate genes and predicted transcription factors (TFs) potentially involved in their regulation. This research provides a starting point for further studies into the molecular mechanisms of IS and may lead to novel intervention strategies targeting neuronal ferroptosis in stroke patients.

## Materials and methods

### Microarray data

We downloaded transcriptome data from IS-related microarrays (coded as GSE61616, GSE35338, and GSE58294) from the Gene Expression Omnibus (GEO) database (https://www.ncbi.nlm.nih.gov/geo/). The GSE61616 dataset was analyzed using Affymetrix Rat 230 2.0 Array, in which we used data expression profile in the infarcted hemispheres at 7 days after Sham operation and MCAO surgery (*n* = 5 per group). We used data from the GSE35338 and GSE58294 for validation. GSE35338 dataset was sourced from male mouse brain tissue after MCAO surgery (*n* = 5) and Sham operation (*n* = 4). GSE58294 dataset contains blood samples from 23 control patients and 69 stroke patients.

### Differential gene expression analysis

We accessed GEO2R (https://www.ncbi.nlm.nih.gov/geo/geo2r/) to compare gene expression data from MCAO-treated and control mice in the GSE61616 dataset, with padj<0.05 and |logFC|>1 as the threshold. The results were presented in the form of a volcano plot and a heatmap. Next, we downloaded 259 genetic regulators of ferroptosis from the FerrDb database (http://www.zhounan.org/ferrdb/index.html), including drivers, suppressors, and markers. Finally, these ferroptosis-related genes were intersected with the DEGs obtained from the analysis of the GSE61616 dataset to identify ferroptosis-related DEGs.

### Functional enrichment analysis

The clusterProfiler package, the org. Hs.eg.db package, and the GOplot package of R software were used to perform Gene ontology (GO) and Kyoto Encyclopedia of Genes and Genomes (KEGG) analysis on ferroptosis-related DEGs using (*p* < 0.05).

### Protein-protein interaction network analysis

We uploaded ferroptosis-related DEGs to the STRING database (http://www/string-db.org/) for PPI network prediction (interaction score >0.4). Nine genes in cluster one were identified by importing analysis results from the STRING database into Cytoscape v.3.7.2, and the Molecular Complex Detection (MCODE) plugin was then used to perform clustering analysis for the ferroptosis-related DEGs (Degree Cutoff = 2, Node Score Cutoff = 0.2, K-Core = 2, and Max. Depth = 100).

### Diagnostic value of ferroptosis-related biomarkers in ischemic stroke

ROC monofactor analysis was performed in the GSE58294 dataset to evaluate the diagnostic value of ferroptosis-related biomarkers in ischemic stroke. Application of the pROC package, ggplot2 for statistical analysis and visualization of ROC monofactor analysis.

### Animals and experimental procedures

C57BL/6 mice (8–10 weeks old, male, weighing 22–25 g) were purchased from Beijing Weitong Lihua Experimental Animal Technology Co. Animals were kept in a specific pathogen-free and environmentally controlled room with free access to standard laboratory food and water and a 12-h light and dark cycle. To mimic IS, a mouse model of middle cerebral artery occlusion/reperfusion (MCAO/R) was established as described previously (Longa et al., 1989). Briefly, mice were anesthetized with isoflurane (5% for induction and 1.5%–2.5% for maintenance). A 6–0 silicone rubber-coated nylon monofilament (Doccol Corporation) was inserted from an incision of the left external carotid artery, then the monofilament was guided into the internal carotid artery through the bifurcation and advanced about 11 mm to occlude the MCA for 120 min. The filament was withdrawn after 120 min to allow reperfusion after re-anesthesia. Sham-operated mice went through the same procedure except for embolization. We dissolved DEX with saline to a concentration of 5 μg/ml. Each mouse was injected intraperitoneally twice with DEX (50 μg/kg) dissolved in saline immediately after reperfusion and 120 min after reperfusion (Li et al., 2020). After 24 h of reperfusion, the Zea Longa scoring method was used to score the MCAO/R model. Mice scoring 2 or three were considered to suffer from IS. The scoring criteria were as follows: 0 (normal or no neurological deficit perceivable), 1 (inability to fully stretch the front paw on the paralyzed lateral), 2 (mice turned in circles towards the paralyzed side), 3 (slouching towards the paralyzed side), and 4 (loss of consciousness without automatic walking).

The animals were divided into three groups: SHAM, MCAO, and MCAO + DEX. The animal study was reviewed and approved by the Animal Care and Use Committee of the Second Affiliated Hospital of Harbin Medical University (Approval No. SYDW 2021–086). All invasive operations were performed after adequate anesthesia in strict compliance with the National Institutes of Health and the Guiding Principles for the Protection and Use of Laboratory Animals.

### Infarct volume measurement

After 24 h of reperfusion, mice were decapitated under deep isoflurane anesthesia. Brains were quickly removed and snap-frozen for 10 min, cut into thin slices of approximately 1 mm along the coronal plane, stained with 2% 2,3,5-triphenyltetrazolium chloride (TTC), incubated at 37°C for 15 min with turning to ensure even staining, fixed in 4% paraformaldehyde overnight, and photographed. Infarct areas were measured using Image-Pro Plus software (Version 6.0, Media Cybernetics, Bethesda, MD, United States). Infarct volume (compensated for brain edema) was calculated as:

(Contralateral hemisphere area−ipsilateral hemisphere without infarct)/contralateral hemisphere area

### TdT-mediated dUTP nick end labeling

The TUNEL method was performed to visualize the 3′-OH ends of DNA fragments in dead cells for paraffin-embedded brain slides according to the manufacturer’s protocol (Roche, Germany). The results are quantified as (TUNEL-positive cells)/(total cells) ×100%.

### Neurobehavioral analysis

Neurobehavioral analysis was performed in a double-blinded manner after MCAO. Sensorimotor deficits were assessed by the rotarod test, the corner test, and the adhesive removal test, performed at 1 day, 3 days, 5 days, 7 days, and 14 days after MCAO. Rotarod test was carried out as described previously after 3 days of adaptation training (Wang et al., 2014; Xu et al., 2020). Briefly, mice were placed on a rotating drum with speed from 4 to 40 rpm during a 5-min period. Each mouse was tested three times per day, with an intermission of at least 15 min between tests. Latency to fall off the rotating rod was recorded. Data were expressed as mean values from the three trials. The adhesive removal test was performed according to previous literature (Bouet et al., 2009; Qu et al., 2021). The test was carried out with 2 × 3 mm tape. Adhesive tapes were applied to the ipsilateral or contralateral forepaw of the mouse to evaluate the sensory and motor function of mice. Time is recorded when the mouse perceives the sticker attached to the foot and removes it as time-to-contact and time-to-remove. The amount of time-to-contact and time-to-remove for the mouse was measured up to 120 s. The corner test is a widely used functional assessment for unilateral sensorimotor cortical damage (Yu et al., 2019). Two cardboard plates (30 cm × 20 cm) were attached at a 30° angle in a home cage. Each mouse was placed between the two plates and allowed to move freely to the corner. The number of times the mice turned to the left in 10 trials were recorded. Only mice without a preference for turning left and right in the pre-training were included. Normal mice make about equal left and right turns in their exploratory turning behavior. After the ischemic and reperfusion insult to the sensorimotor cortex, mice showed biased turns consistent with the side of their brain damage.

### Enzyme linked immunosorbent assay

Mice were sacrificed after 24 h of reperfusion, and blood samples collected by cardiac puncture were stored at −80°C before analysis. Serum levels of TNF-α and IL-6 were determined by commercial ELISA kits (Mouse TNF-α and IL-6 ELISA Kit, Jingkang, China), following the manufacturer’s instructions.

### Detection of MDA levels

Lipid peroxidation in the ischemic penumbra area was assessed using an MDA assay kit (S0131, Beyotime Institute of Biotechnology, China), in accordance with the manufacturer’s instructions. Absorbance measurements were made at 532 nm.

### GSSG content detection

Oxidized glutathione disulfide (GSSG) levels were measured in tissue homogenates obtained from the penumbra area using a GSSG detection kit (S0053, Beyotime Institute of Biotechnology). Absorbance measurements were made at 412 nm.

### Western blotting

Protein was extracted from the penumbra area of mice with RIPA lysis buffer. Primary antibodies against GPX4 (Abcam, ab125066), HIF1A (CST, #36169), and β-actin (ABclonal, AC026) were used. After washing, incubate the membranes with an anti-rabbit secondary antibody (Abcam, ab6721).

### Extraction of RNA and qRT-PCR

We used trizol reagent to extract RNA from ischemic penumbra brain tissue. cDNA was then synthesized with the First Strand cDNA Synthesis Kit (Code No. FSK-101, Toyobo, Osaka, Japan) on a thermal cycler (BIO-RAD S1000). Quantitative RT-PCR (qRT-PCR) was performed using AceQ Universal SYBR qPCR Master Mix (Vazyme, Nanjing, China). Target genes’ mRNA levels were normalized to endogenous GAPDH expression and quantified by the 2-△△ct method. Primer sequences were as follows:

Gapdh-F: 5′-AGG​TCG​GTG​TGA​ACG​GAT​TTG.

Gapdh-R: 5′-TGT​AGA​CCA​TGT​AGT​TGA​GGT​CA;

*HMOX1*-F: 5′-GCC​CCA​CCA​AGT​TCA​AAC​AG.

*HMOX1*-R: 5′-GCT​CCT​CAA​ACA​GCT​CAA​TGT;

*STAT3*-F: 5′-TGG​GCT​AAA​TTC​TGC​AAA​GAA​AAC.

*STAT3*-R: 5′-GGC​TTT​GTG​CTT​AGG​ATG​GC;

*CYBB*-F: 5′-TGG​AAA​CCC​TCC​TAT​GAC​TTG​G.

*CYBB*-R: 5′-AAA​CCG​AAC​CAA​CCT​CTC​ACA​AA;

*TLR4*-F: 5′-GCT​TGA​ATC​CCT​GCA​TAG​AGG​TAG.

*TLR4*-R: 5′-TGT​CAT​CAG​GGA​CTT​TGC​TGA​G.

TNFα-F: 5′- GTA​GCC​CAC​GTC​GTA​GCA​AA.

TNFα-R: 5′- ACA​AGG​TAC​AAC​CCA​TCG​GC.

Il6-F: 5′- CGT​GGA​AAT​GAG​AAA​AGA​GTT​GTG​C.

Il6-R: 5′- GGT​ACT​CCA​GAA​GAC​CAG​AGG​A.

### Construction of an mrna-mirna-lncrna interaction network

We used a combination of five databases (miRDB, miRmap, miRWalk, RNA22, and TargetScan) to predict miRNAs with potential regulatory activity on the candidate genes. LncRNA prediction of selected miRNA was performed *via* starBase v2.0 (https://starbase.sysu.edu.cn/) with the following screening criteria: mammalian, human h19 genome, strict stringency (≥5) of CLIP-Data, with or without degradome data. We then created a competing endogenous RNAs (ceRNA) regulatory network for DEGs using Cytoscape.

### Transcription factor prediction

After retrieving high-confidence and experimentally validated gene-TF pairs *via* the NetworkAnalyst platform and JASPAR database, Cytoscape software was used to create a TF-based regulatory network of candidate ferroptosis-related genes.

### Statistical analyses

Statistical analyses were done using SPSS 25.0 (IBM, Armonk, NY, United States), Prism 6.01 (GraphPad, San Diego, CA, United States), and R. Data were displayed as mean ± SD. The Student’s t-test was used for comparison between two groups. One-way ANOVA was employed for comparison between multiple groups, and the post hoc multiple comparisons least significant difference and Student–Newman–Keuls model were selected for analyses, *p* < 0.05 was considered significant.

## Results

### Identification of ferroptosis-related DEGs after IS

**FIGURE 1:**
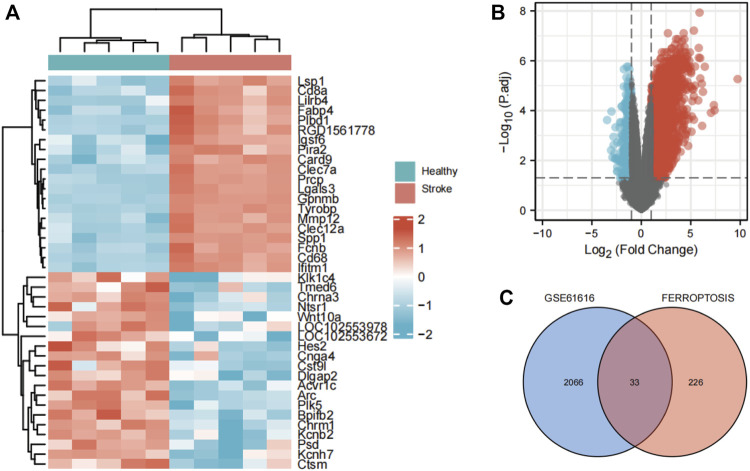
Identification of ferroptosis-related DEGs in IS. **(A)** Heatmap of DEGs between the MCAO cohort and the sham cohort in the GSE1 dataset. **(B)** Volcano plots of DEGs. **(C)** Venn diagram of DEGs from the two datasets.

Using defined criteria, we identified 2099 DEGs between control and IS samples in the GSE61616 dataset. Heatmaps of all these DEGs showed excellent discrimination between IS and control samples and volcano plots of the top 20 genes that were highly expressed and the top 20 genes that were lowly expressed in the MCAO group compared to the SHAM group (Figure Figure1 A, B). We also acquired a dataset that included 259 genes from the Ferroptosis Database (FerrDb). Venn diagram analysis of these two gene sets identified 33 ferroptosis-related DEGs (Figure 1C), including 28 up-regulated and five down-regulated genes (Table 1).

**TABLE 1:** Ferroptosis-related differentially expressed genes.

Gene symbol	Name	logFC	adj.P.Val	Id
RGS4	Regulator of G protein signaling 4	−1.53	3.15E-02	1368505_at
GLS2	Glutaminase 2	−1.26	1.47E-02	1370375_at
FBXW7	F-box and WD repeat domain containing 7	−1.05	8.85E-03	1379332_at
VLDLR	Very low density lipoprotein receptor	−1.02	9.90E-05	1392924_at
DUSP1	Dual specificity phosphatase 1	−1.01	9.08E-03	1368146_at
SAT1	Spermidine/spermine N1-acetyltransferase 1	1.03	1.09E-04	1371774_at
*STAT3*	Signal transducer and activator of transcription 3	1.13	5.53E-04	1370224_at
FANCD2	FA complementation group D2	1.25	4.53E-05	1378240_at
HERPUD1	Homocysteine inducible ER protein with ubiquitin like domain 1	1.32	6.45E-03	1367741_at
RIPK1	Receptor interacting serine/threonine kinase 1	1.34	8.90E-05	1371529_at
BID	BH3 interacting domain death agonist	1.42	1.48E-04	1377759_at
GDF15	Growth differentiation factor 15	1.45	1.20E-02	1370153_at
IL6	Interleukin 6	1.46	9.28E-03	1369191_at
LPCAT3	Lysophosphatidylcholine acyltransferase 3	1.52	7.59E-05	1376813_at
ANO6	Anoctamin 6	1.60	1.35E-03	1372624_at
HIC1	HIC ZBTB transcriptional repressor 1	1.72	3.68E-04	1381942_at
LAMP2	Lysosomal associated membrane protein 2	1.76	1.20E-05	1370010_at
NFE2L2	Nuclear factor, erythroid 2 like 2	1.86	2.67E-06	1367826_at
TXNIP	Thioredoxin interacting protein	1.96	1.57E-03	1371131_a_at
SLC1A5	Solute carrier family 1 member 5	2.04	1.25E-05	1371040_at
CD44	CD44 molecule (Indian blood group)	2.15	1.13E-05	1368921_a_at
AURKA	Aurora kinase A	2.21	2.65E-05	1376039_at
ATF3	Activating transcription factor 3	2.30	1.16E-03	1369268_at
CDKN2A	Cyclin dependent kinase inhibitor 2 A	2.40	1.37E-03	1369194_a_at
GCH1	GTP cyclohydrolase 1	2.42	1.31E-04	1387221_at
PLIN2	Perilipin 2	2.52	7.28E-05	1382680_at
TGFBR1	Transforming growth factor beta receptor 1	2.88	8.21E-07	1376636_at
*TLR4*	Toll like receptor 4	2.94	1.08E-05	1387982_at
HSPB1	Heat shock protein family B (small) member 1	3.05	2.61E-05	1367577_at
ALOX12	Arachidonate 12-lipoxygenase, 12 S type	3.09	1.49E-02	1380636_at
*HMOX1*	Heme oxygenase 1	3.32	1.12E-05	1370080_at
CAPG	Capping actin protein, gelsolin like	4.41	7.50E-06	1388460_at
*CYBB*	Cytochrome b-245 beta chain	4.88	1.08E-05	1379344_at

### GO and KEGG analysis of ferroptosis-related DEGs

**FIGURE 2:**
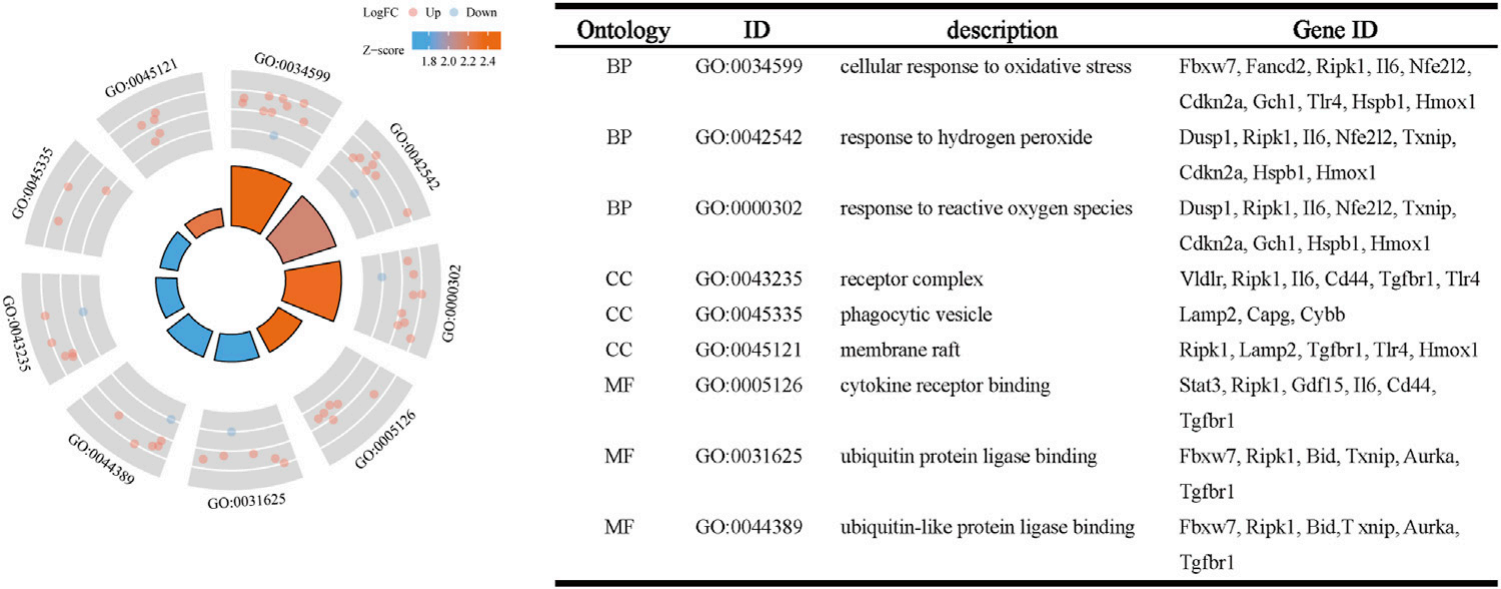
Gene ontology enrichment analysis of ferroptosis-related DEGs.

**FIGURE 3:**
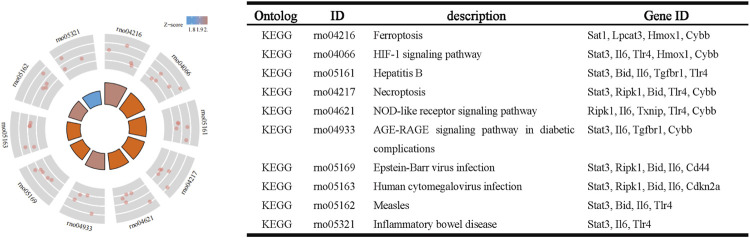
Kyoto Encyclopedia of Genes and Genomes pathway analysis of ferroptosis-related DEGs.

The R package clusterProfiler was used to conduct GO and KEGG pathway enrichment analysis of the 33 ferroptosis-related DEGs. On GO analysis, for the biological process (BP), cell component (CC), and molecular function (MF) categories, these DEGs were mainly enriched in ‘cellular response to oxidative stress,’ ‘receptor complex,’ and ‘cytokine receptor binding,’ respectively (Figure 2). On KEGG pathways analysis, the ferroptosis-related DEGs were mainly enriched in ‘ferroptosis’, followed by ‘HIF-1 signaling pathway’ (Figure 3). This finding supports the notion that HIF-1 signaling is intrinsically linked to the occurrence of ferroptosis in IS.

### PPI analysis of ferroptosis-related DEGs

**FIGURE 4:**
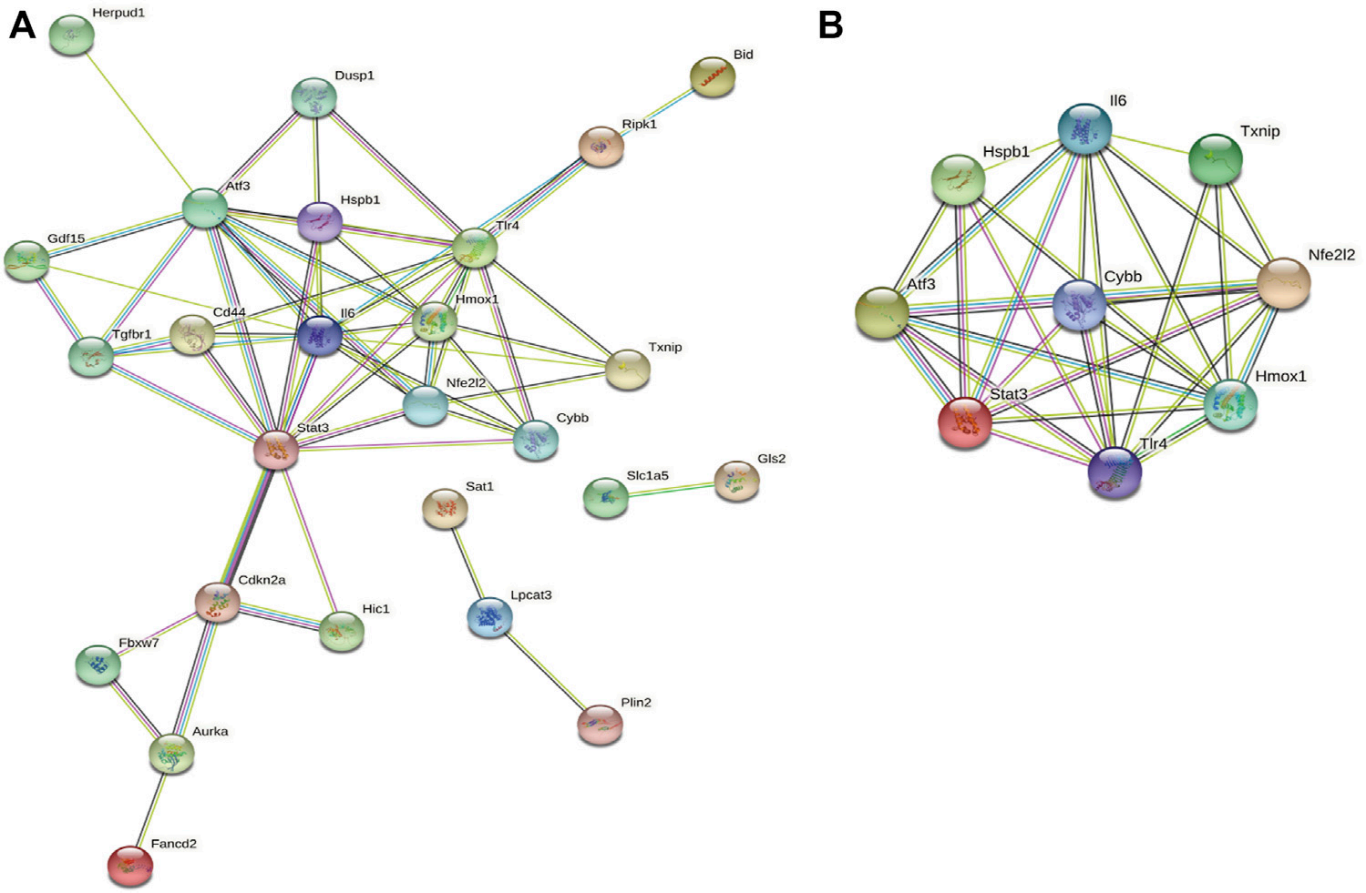
PPI analysis of ferroptosis-related DEGs. **(A)** PPI network of ferroptosis-related DEGs. **(B)** Gene clustering based on the MCODE algorithm.

Next, we accessed the STRING database and constructed, a PPI network for the 33 ferroptosis-related DEGs using Cytoscape software. This network included 33 nodes and 64 edges (Figure 4A). MCODE was used to explore the most significant cluster (cluster one; containing nine genes). The nine genes in cluster one were then incorporated into the PPI network map (Figure 4B).

### Functional enrichment analysis of cluster one

**FIGURE 5:**
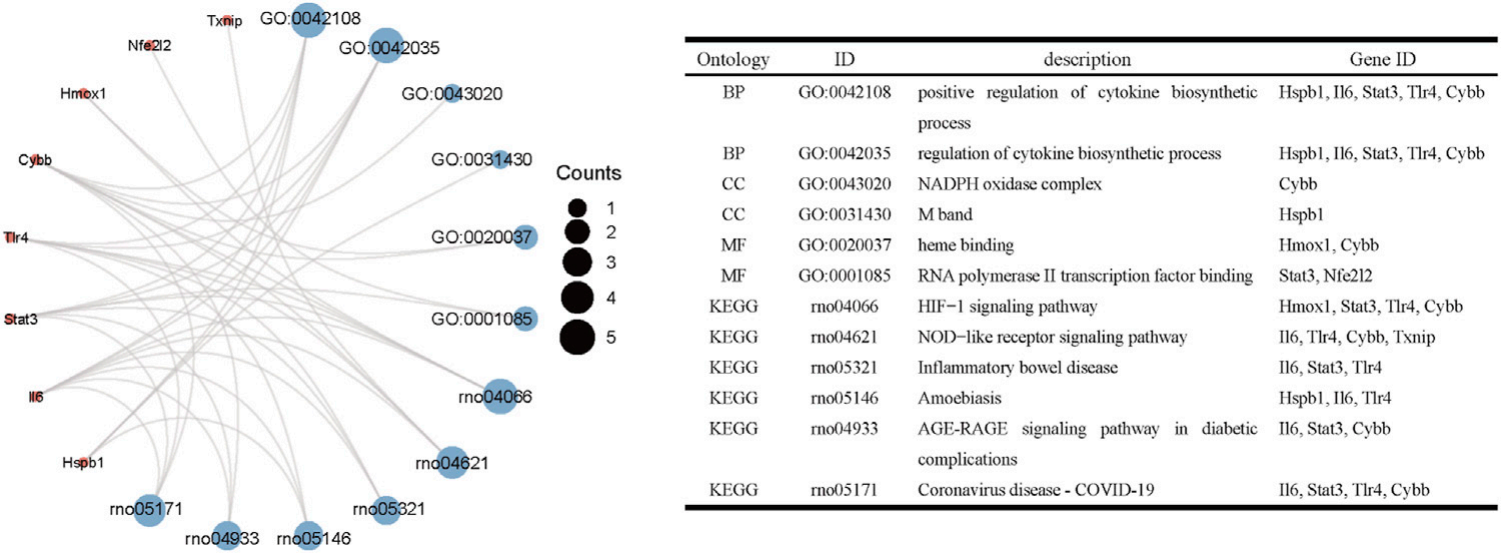
Functional enrichment analysis of genes in cluster 1.

Based on GO and KEGG analysis, we found that four out of the nine genes in cluster1 (*HMOX1*, *STAT3*, *CYBB*, and *TLR4*) were enriched in the HIF-1 signaling pathway. These genes were then selected as candidate genes for further analysis (Figure 5).

### Verification of the expression of the four candidate genes in IS

**FIGURE 6:**
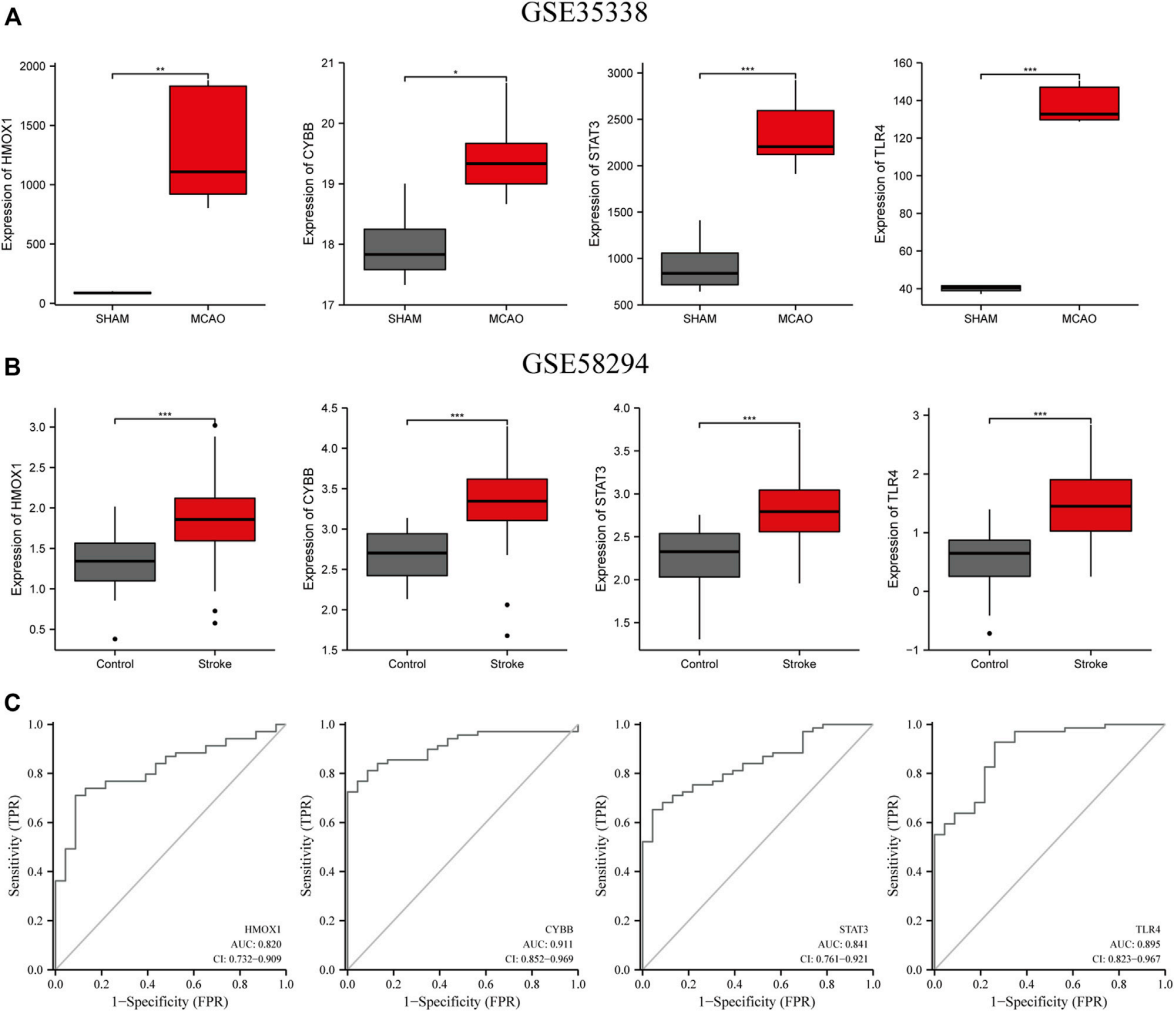
Verification of the expression levels of the four candidate genes (HMOX1, CYBB, STAT3, TLR4) **(A)** in MCAO and sham mice brain samples in the GSE35338 dataset **(B)** and in the peripheral blood of control and IS patients in GSE58294. **(C)** The AUC of ferroptosis-related biomarkers in the diagnosis of IS in ROC monofactor analysis in the GSE58294 dataset. ***p<0.001, **p <0.01, *p<0.05. ***p<0.001, **p <0.01, *p<0.05.

Next, we validated the expression levels of the four candidate ferroptosis-related genes by assessing their expression in two different IS-related datasets (GSE35338 and GSE58294; Figures 6A,B). The expression levels of all four genes were significantly upregulated in samples from the IS group compared to those of the sham-operated group. These results, which were consistent with those obtained from the GSE61616 dataset, further suggest the importance of these four ferroptosis-related genes in IS.

### The diagnostic value of four candidate genes in IS

ROC univariate analysis was performed in the GSE58294 dataset to assess the diagnostic value of four candidate genes for IS. As a result, the accuracy of *HMOX1*, *STAT3*, *CYBB*, and *TLR4* in diagnosing IS in the GSE58294 dataset was 0.820, 0.841, 0.911, and 0.895, respectively (Figure 6C).

### DEX alleviated cerebral ischemic/reperfusion injury in a mouse model of IS

**FIGURE 7:**
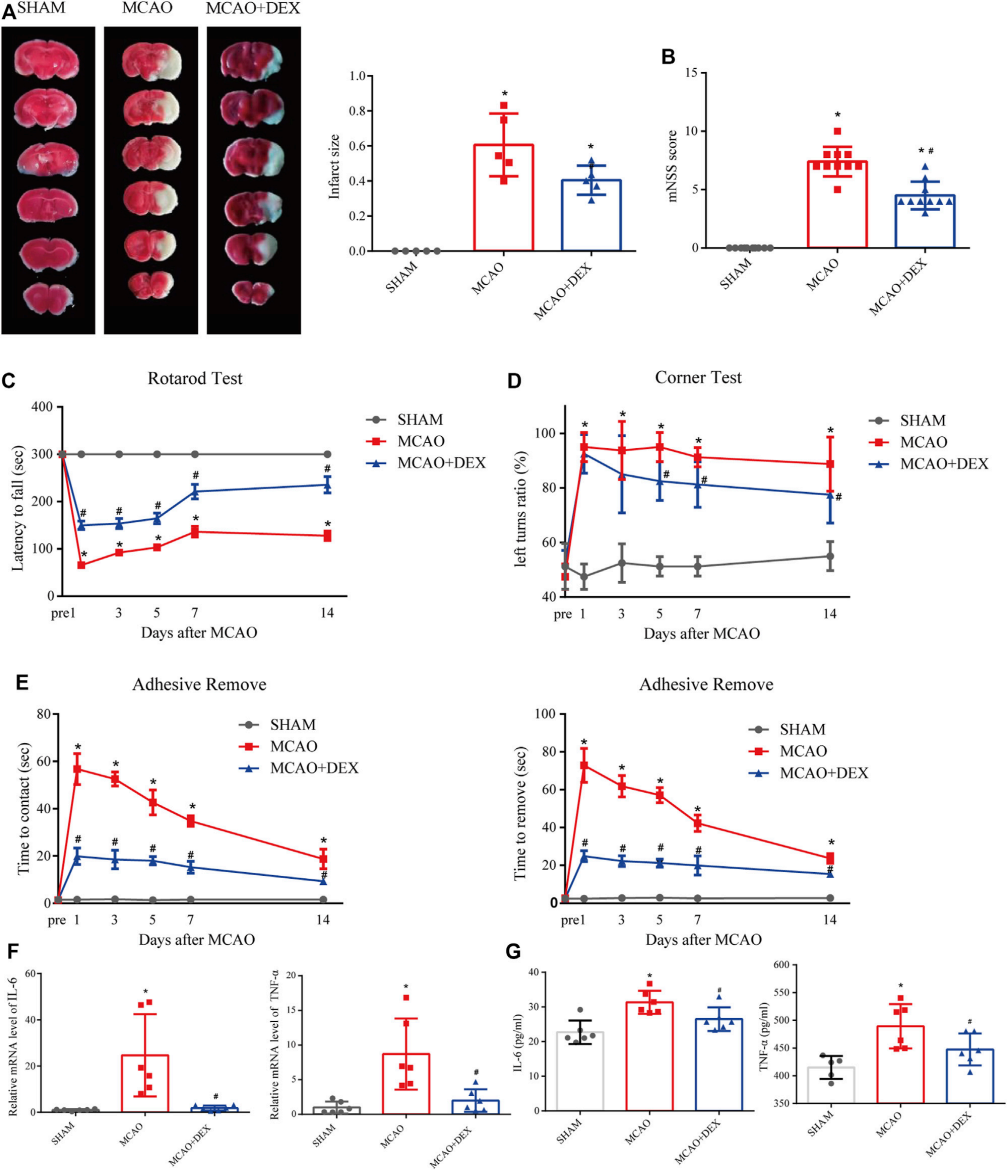
**(A)** Representative TTC staining in comparable sections from the three mice groups and quantification of infarct volume (*n* = 5). **(B)** Modified neurological severity (mNSS) scores for mice in the three experimental groups (*n* = 10). On different days after ischemic stroke, **(C)** the time for the mice to contact or remove the sticker was recorded, **(D)** the latency to fall off the rotating rod, and **(E)** the turning bias in the corner test. *n* = 8 per group. **(F)** Brain levels of IL-6 **(E)** and TNF-α. *n* = 6 per group **(G)** Serum levels of IL-6 (*n* = 6) and TNF-α (*n* = 5 for SHAM group, and *n* = 6 for MCAO and MCAO + DEX group). Data are presented as the mean ± SD. *p<0.05 vs. SHAM group, #p<0.05 vs. MCAO group.

We next investigated the effect of dexmedetomidine (DEX), a highly selective alpha-2 adrenoceptor agonist with anti-ferroptosis action, in a mouse model of MCAO-induced IS. After 24 h of reperfusion, we determined infarct size by staining brain sections with TTC. As shown in Figure 7A, infarct size was significantly attenuated by DEX administration. In addition, neurological deficits in the MCAO + DEX group were significantly milder than those in the MCAO group (Figure 7B). To further assess the sensorimotor function of the mice, we applied the rotarod test, the corner test, and the adhesive removal test. IS induced severe sensorimotor impairment in mice, which was manifested as decreased latency to fall off the rotating rod (Figure 7C), increased number of left turns in the corner test (Figure 7D), and a prolonged time to contact and remove the tape in the adhesive removal test (Figure 7E). Interestingly, DEX showed a protective effect on sensorimotor dysfunction by increasing the right limb use and correcting for the turning bias in 5, 7 and 14 days after MCAO. However, no turn bias was observed in the acute phase of IS (1 and 3 days after MCAO). To assess whether the beneficial effects of DEX on IS are related to the inhibition of inflammatory processes, we quantified through ELISA serum and brain levels of pro-inflammatory cytokines. A prominent reduction in TNF-α and IL-6 levels in the brain was noted in the MCAO + DEX group compared with the MCAO group (Figure 7F). And there is a mild decrease in serum levels of TNF-α and IL-6 in the MCAO + DEX group compared with the MCAO group (Figure 7G). These findings are thus consistent with previous evidence indicating that dexmedetomidine alleviates IS severity in rodent models of CIRI.

### DEX alleviated ferroptosis and regulated candidate genes

**FIGURE 8:**
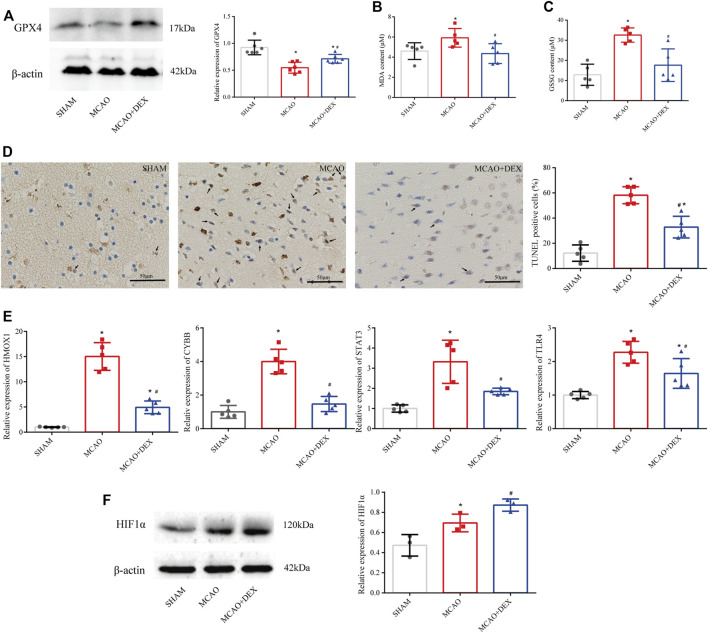
**(A)** Western blotting analysis of GPX4 in the penumbra area (*n* = 6 per group). **(B)** MDA levels in brain tissues (*n* = 5 per group). **(C)** GSSG levels in brain tissues (*n* = 6 per group). **(D)** TUNEL staining in SHAM, MCAO and DEX + MCAO mice (Scale bars = 50 μm) and quantification of the number of TUNEL+ cells (*n* = 3). **(E)** Relative mRNA levels of HMOX1, CYBB, STAT3, and TLR4 in mice brain by qRT-PCR (*n* = 5). **(F)** Western blotting analysis of HIF1α in the penumbra area (*n* = 3). Data are presented as the mean ± SD. *p<0.05 vs. SHAM group, #p<0.05 vs. MCAO group.

Western blotting analysis of brain tissue revealed markedly higher levels of GPX4 in DEX-treated mice compared to the untreated MCAO mice (Figure 8A). We next analyzed the impact of DEX on oxidative stress, a primary trigger of ferroptosis, by measuring the contents of MDA and GSSG in the penumbra region of the infarcted tissue. Results showed a significant reduction in both MDA and GSSG levels following administration of DEX (Figures 8B,C). Because ferroptosis has TUNEL positivity as described previously (Ueta et al., 2012), we used TUNEL staining *in vivo* as a potential marker of ferroptosis and observed that TUNEL-positive cells increased in the brain of MCAO mice. However, the proportion of TUNEL-positive cells in the MCAO + DEX group was lower (Figure 8D). Using Western blotting analysis and qRT-PCR analysis, we verified whether DEX treatment influenced the expression of HIF1α and the four ferroptosis-related candidate genes selected through our bioinformatics analysis. Results showed that after IS induction, brain *HMOX1*, *STAT3*, *CYBB*, and *TLR4* mRNA levels were dramatically decreased in DEX-treated mice (Figure 8E). Protein expression levels of HIF1α in brain tissue were elevated after MCAO and had higher expression in the DEX group (Figure 8F). These findings provide robust evidence that DEX inhibits CIRI-induced ferroptosis by blunting HIF-1 signaling through regulation of *HMOX1*, *STAT3*, *CYBB*, and *TLR4* expression.

### Construction of a mRNA -miRNA-lncRNA interaction network

**FIGURE 9:**
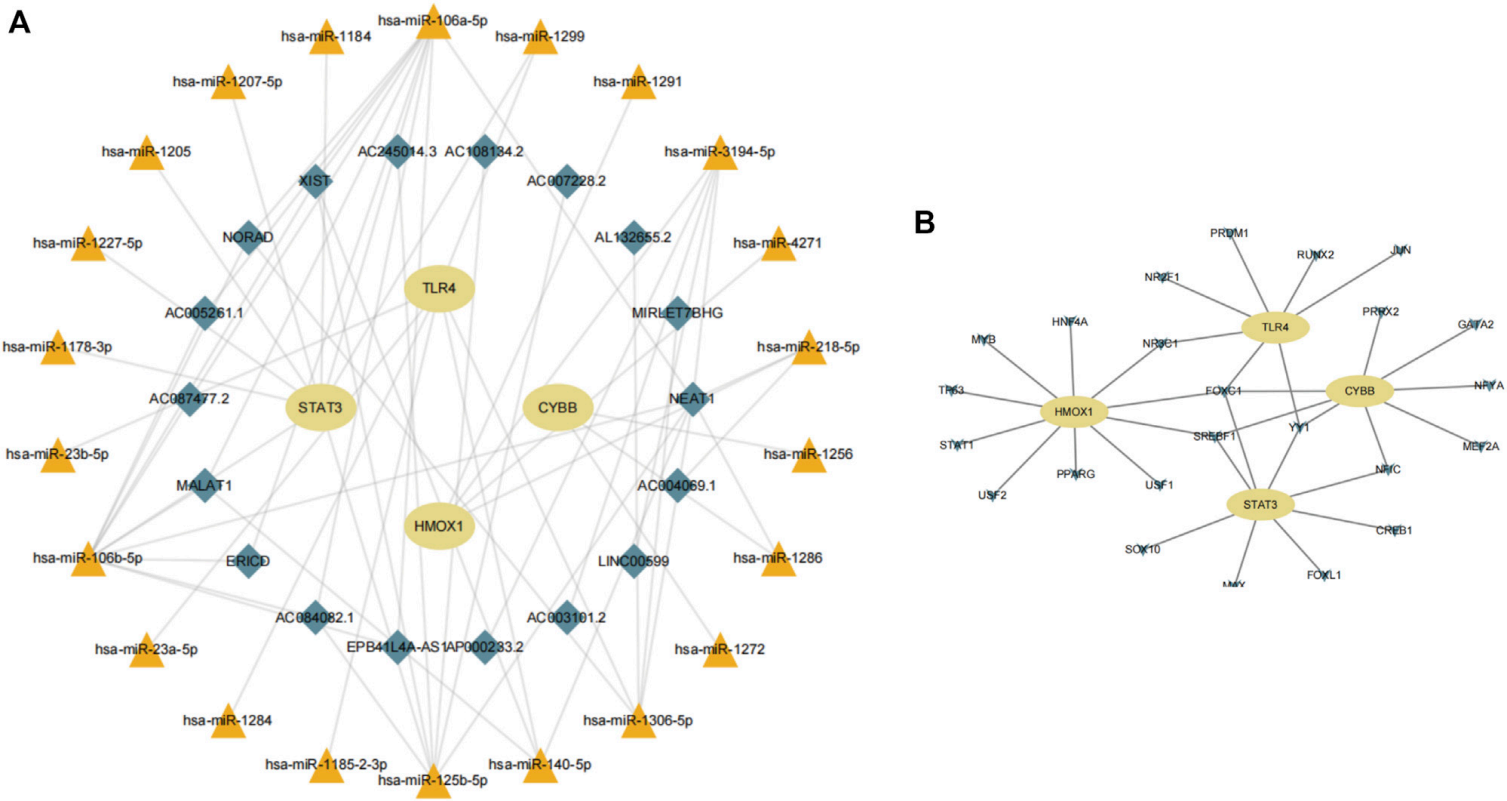
**(A)** Construction of a gene-miRNA-lncRNA interaction network. **(B)** Identification of potential TFs.

Next, we consulted the miRDB, miRmap, miRWalk, RNA22, and TargetScan databases to identify potential regulatory miRNAs for the *HMOX1*, *STAT3*, *CYBB*, and *TLR4* transcripts. Among a total of 22 miRNAs retrieved, hsa-miR-106a-5p and hsa-miR-106b-5p ranked first, while hsa-miR-1299 was found to bind to more than one mRNA. Subsequently, for the 22 miRNAs predicted, analysis on the starBase platform identified 18 potentially interacting lncRNAs. Based on the above analysis, a ferroptosis-related mRNA-miRNA-lncRNA interaction network is presented in Figure 9A.

### Identification of TFs potentially influencing the expression of ferroptosis-related genes in IS

Using data from NetworkAnalyst, we finally created an interaction network with 32 connections featuring the four candidate genes and 24 TFs predicted to regulate their expression (Figure 9B). Among the predicted TFs, NR3C1, FOXC1, SREBF1, YY1, and NF1C were shown to regulate more than one candidate gene, thus suggesting a high level of interaction within the ferroptosis-related gene-TF network.

## Discussion

Ferroptosis has been widely studied in neoplastic disease, and its role in neuronal cell death is becoming well established (Lu et al., 2017; Datta et al., 2020). Previous research showed that ferroptosis aggravates the outcome of CIRI; hence, its inhibition may represent a novel therapeutic direction (Tuo et al., 2017). However, the molecular events and signaling pathways associated with ferroptosis induction in IS have not been thoroughly investigated.

The HIF-1 signaling pathway is activated under hypoxic conditions and acts as a master regulator of multiple pathological processes in IS by modulating glucose metabolism, angiogenesis, erythropoiesis, and cell survival (Pan et al., 2021). A rapid and transient increase in the activation of the HIF-1 signaling pathway in brain endothelial cells is responsible for blood-brain barrier disruption, especially during severe and sustained hypoxia (Yeh et al., 2007; Yan et al., 2011). A previous study confirmed that activation of the HIF-1 signaling pathway is closely related to ferroptosis (Li et al., 2020). Therefore, HIF-1 signaling activation may play a crucial role in ferroptosis induction during IS.

In the present study, we identified four essential ferroptosis-related genes involved in the pathogenesis of IS and predicted, for these candidate genes, a regulatory non-coding RNA network as well as several putative TFs. By intersecting the GSE61616 and FerrDb dataset, we identified 33 ferroptosis-related DEGs, including 28 upregulated and five downregulated genes. Functional enrichment analysis of these DEGs showed that they were mainly clustered in the HIF-1 signaling pathway. Then, nine hub genes, *Il6*, *TXNIP, NFE212, HMOX1, TLR4, STAT3, ATF3, HSPB1*, and *CYBB*, were identified by PPI and MCODE analysis in cluster 1. Notably, four of the nine hub genes (*HMOX1, TLR4, STAT3,* and *CYBB*) were also enriched in the HIF-1 signaling pathway, a finding that suggested their central involvement in ferroptosis-mediated neuronal death in CIRI.

*HMOX1* encodes heme oxygenase 1, an enzyme involved in the catabolism of cellular heme induced by various pro-oxidant and inflammatory stimuli. Animal studies indicated that HO-1 expression in the brain is induced by stroke and plays, *via* activation of Nrf2, a protective role by exerting antioxidant, antiapoptotic, anti-inflammatory, and vasorelaxant effects (Bereczki et al., 2018). Studies have demonstrated that HO-1 expression in brain tissue is significantly increased within 24 h after IS, which is consistent with the results of our bioinformatics analysis and returns to baseline levels after several months. In our mouse model of IS, we verified that the mRNA level of *HMOX1* in the penumbra region was significantly increased compared to non-infarcted brain tissue from sham-operated mice. In addition, we found that GPX4 protein levels were downregulated in affected brain tissue from mice with MCAO-induced IS. Recent research on cognitive impairment after CIRI concluded that HO-1 levels were negatively correlated with the extent of ferroptosis 14 days after reperfusion (Fu et al., 2022). Interestingly, our animal experiments showed that DEX treatment rescued GPX4 expression in MCAO mice. Although this may be related to the fact that in this study DEX treatment mildly decreased HMOX-1 mRNA expression, the specific mechanisms need to be confirmed by further experiments.

*STAT3* belongs to the signal transducer and activator of the transcription family of proteins. Interestingly, *STAT3* activation during stroke was shown to exert pro-inflammatory and anti-inflammatory effects on different cell types and stages (Qi and Yang, 2014). Upregulation of *STAT3* in microglia was shown to exacerbate CIRI by promoting the expression of various pro-inflammatory factors, including TNF-α and IL-6 (Hu et al., 2017; Bousoik and Montazeri Aliabadi, 2018). In the present work, the upregulation of *STAT3* mRNA observed in the ischemic penumbra area of MCAO mice was consistent with the results of the aforementioned study, and *STAT3* levels in peripheral blood also have considerable diagnostic efficacy for acute ischemic stroke (AIS). Of note, recent studies indicated that *STAT3* expression critically regulates ferroptosis in various diseases, including acute lung injury and lymphoma (Qiang et al., 2020; Schmitt et al., 2021). However, the impact of *STAT3* signaling on IS-related ferroptosis remains unexplored. Thus, although further confirmatory experiments are warranted, the present findings suggest that *STAT3* might be an important therapeutic target to inhibit ferroptosis in IS.

The *CYBB* gene encodes the cytochrome b-245 beta chain, also known as NADPH oxidase 2 (NOX2). This enzyme is one of the major ROS-producing isoforms in IS and is closely associated with poor outcomes. Consistent with the results of our study, *CYBB* was found to be significantly upregulated in brain tissue from mice with experimental IS(Li et al., 2014). In addition, in our previous study, we observed that silencing of *CYBB* in microglia notably reduced neuronal apoptosis in a co-culture system (Zeng et al., 2018). These findings are thus consistent with evidence indicating that *CYBB* is an important ferroptosis driver and hence a promising therapeutic target to reduce ferroptosis during stroke.

The *TLR4* gene encodes a member of the Toll-like receptor (TLR) family of proteins, which show increased expression during stroke through multiple pathways (Caso et al., 2007). Recent studies have identified the possible involvement of *TLR4* in post-stroke neuroinflammation, specifically in neutrophil differentiation and CNS infiltration, thus highlighting the regulation of *TLR4* at the peripheral level in stroke (García-Culebras et al., 2019). The present study reported the upregulation of *TLR4* expression in peripheral blood of ischemic stroke patients from the GEO database and verified that in brain tissue of MCAO mice, which is consistent with the results of previous studies. As a ferroptosis driver, its role in hypoxic-ischemic brain damage-induced ferroptosis and brain homeostasis has been validated by Zhu’s study (Zhu et al., 2021). As expected, in the present study, GPX4 levels were negatively correlated with *TLR4* mRNA levels in brain tissue from MCAO mice. Hence, we speculate that *TLR4* may aggravate CIRI by promoting ferroptosis and oxidative stress. However, further research is needed to elucidate the mechanisms by which *TLR4* triggers ferroptosis in IS.

The results of our bioinformatics analysis were corroborated in a mouse model of MCAO-induced IS. In addition, we evaluated the effect of DEX, a sedative agent with analgesic and anti-ferroptosis actions, on both IS severity and the expression of the above four ferroptosis-related genes (Gao et al., 2019; Ross and Siegel, 2021; She et al., 2021). In line with previous findings, qRT-PCR assays showed *HMOX1*, *CYBB*, *STAT3*, and *TLR4* were significantly upregulated after IS induction. In our MCAO model, DEX reversed this effect. Recent studies have found that DEX exerts a protective effect by modulating the HIF-1 signaling pathway, improving mitochondrial function, and inhibiting ferroptosis in endothelial cells (She et al., 2021). However, whether DEX attenuates ferroptosis in IS and whether this effect is associated with inhibition of HIF-1 signaling has not been so far determined. Our study demonstrated that DEX significantly reduced oxidative stress in the brain and reduced circulating levels of pro-inflammatory cytokines in MCAO mice. Furthermore, DEX also reversed the downregulation of GPX4, a key negative regulator of ferroptosis, and the upregulation of ferroptosis-related gene expression induced by IS. Therefore, these findings suggest plausible mechanisms by which DEX therapy may reduce ferroptosis-mediated neuronal death in stroke.

To further investigate the factors that may affect the expression of our candidate genes, we identified TFs that may bind to their promoter regions. In addition, based on the four candidate genes, an mRNA-miRNA-lncRNA network was constructed. Of the 22 miRNAs obtained by targeting the four candidate genes, hsa-miR-1299 was found to bind to more than one mRNA. Studies on hsa-miR-1299 unmasked its inhibitory role on tumor cell proliferation (Zhang et al., 2020) but have not addressed its potential role in IS. However, the top-ranking predicted miRNAs were hsa-miR-106a-5p and hsa-miR-106b-5p. miR-106 is associated with microglial activation and neuroinflammatory diseases (Liu et al., 2021). Given the complexity of its regulatory network, miR-106 may represent an essential target for the regulation of stroke. LncRNAs represent promising targets for the diagnosis and treatment of various diseases. These non-coding RNAs have diverse functions and can interact with and regulate the expression of various endogenous molecules, including miRNAs(Bao et al., 2021; Bridges et al., 2021). In the present study, nine out of the 22 retrieved miRNAs were known targets of lncRNAs. Of these, NEAT1 may regulate up to eight miRNAs. Based on the neuroprotective role of NEAT1 (Ni et al., 2020; Zhou et al., 2022) we propose that NEAT1 may regulate, via a ceRNA network, the expression of ferroptosis-related genes in IS through mutual interactions with multiple miRNAs among the 24 TFs identified, FOXC1, SREBF1, and YY1 were found to target three of the four candidate genes. The upregulation of FOXC1 has been shown to alleviate neuroinflammation in mice with ischemic brain injury (He et al., 2021). Interestingly, YY1-induced upregulation of NEAT1 led to inflammatory damage following oxygen-glucose deprivation and reperfusion injury in microglia (Chen et al., 2019; Han and Zhou, 2019), thus contradicting the widely recognized neuroprotective role of NEAT. Whether this effect is or is not cell-type specific requires further investigation.

## Conclusion

We identified four candidate ferroptosis-related genes, *HMOX1*, *CYBB*, *STAT3*, and *TLR4*, which may be involved in neuronal ferroptosis in CIRI. In addition, we showed that DEX treatment reduced infarct volume, improved sensorimotor function, and downregulated the expression of ferroptosis-related genes in mice with MCAO-induced IS. These genes may thus serve as new therapeutic targets to prevent or attenuate ferroptosis and thus improve prognosis in stroke patients. In addition, we predicted through bioinformatics analyses a set of miRNAs, lncRNAs, and TFs that may regulate the above candidate genes. The results of this study may broaden our understanding of IS and provide new concepts for its treatment. However, there are some limitations in the present work. Due to the disease characteristics, we could not apply gene expression data from human brain tissue for analysis, nor were we able to collect enough brain tissue from AIS patients to validate our findings. Furthermore, we did not perform experimental validation of the predicted miRNAs, lncRNAs, and TFs, therefore, further verification is required.

## Data Availability

The datasets analyzed for this study can be found in the GEO database (www.ncbi.nlm.nih.gov/geo/). The accession numbers can be found in the article.
